# Self-powered, high response and fast response speed metal–insulator–semiconductor structured photodetector based on 2D MoS_2_†

**DOI:** 10.1039/c8ra05511d

**Published:** 2018-08-06

**Authors:** Xinxin Liu, Feng Li, Minxuan Xu, Junjie Qi

**Affiliations:** State Key Laboratory for Advanced Metals and Materials, School of Materials Science and Engineering, University of Science and Technology Beijing Beijing 100083 People's Republic of China junjieqi@ustb.edu.cn

## Abstract

Here, we firstly fabricated a metal–insulator–semiconductor (MIS) (Pd/Al_2_O_3_/MoS_2_) self-powered photodetector based on MoS_2_, which is sensitive to the illumination of light without any external bias, exhibiting a high responsivity of 308 mA W^−1^. Under bias, it shows a ratio of photocurrent to dark current exceeding 3705, a high photoresponsivity of 5.04 A W^−1^, and a fast response/recovery time of 468 ms/543 ms. The optoelectronic performances of the photodetector are closely related to the insulating layer, which can suppress the dark current of the photodetectors, and prevent strong current drifting and degradation by environmental effects, playing a key role in carrier tunneling. Furthermore, we used a thin HfO_2_ film as the insulating layer to improve the optoelectronics performance of the MIS structured self-powered photodetector, which presented a high responsivity of 538 mA W^−1^ at 0 bias. With an applied bias, it exhibits an on/off ratio up to 6653, a photoresponsivity of 25.46 A W^−1^, and a response/recovery time of 7.53 ms/159 ms. Our results lead to a new way for future application of high performance MIS structured photodetectors based on 2D MoS_2_.

## Abstract 2

A MIS structured self-powered photodetector of Pd/HfO_2_/MoS_2_ was fabricated by inserting a thin insulator, which has a fast response/recovery speed.

## Graphical abstract

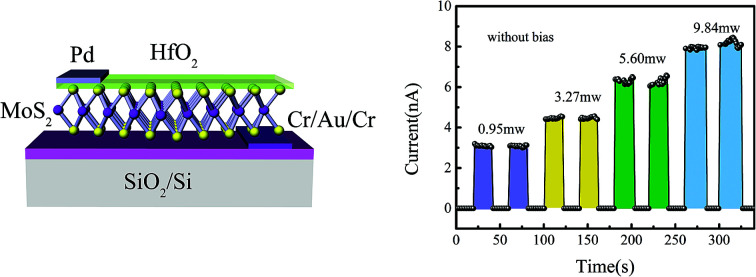

## Introduction

In the past ten years, because of the unique properties of atomically thin two-dimensional layered transition metal dichalcogenides (TMDs), they have drawn more and more attention for their potential applications in future nanoscale electronic/optoelectronic devices.^1–11^ Molybdenum disulfide (MoS_2_), as an important member of TMDs, has been widely studied in recent years. The unique properties of MoS_2_ demonstrate that it is a promising material for optical modulators,^12^ supercapacitors^13^ and highly sensitive photodetectors.^14–17^ To date, many reports on MoS_2_ photodetectors have been published. Zhang H. *et al.*^18^ fabricated a phototransistor based on single-layer MoS_2_; the responsivity can reach as high as 7.5 mA W^−1^ under illumination with a low optical power. However, the responsivity of the MoS_2_ photodetector is still low due to its low carrier mobility, which prevents the successful integration of MoS_2_-based optoelectronic devices. With that consideration, various strategies, including surface modification and structure optimization, have been proposed to enhance the performances of MoS_2_ based optoelectronics, especially photodetectors. Some works reported that designing an asymmetric metal contact formed a strong built-in electric-field to break the symmetry of the built-in electric-field profile in the traditional photodetector channel.^19–22^ Xu *et al.*^22^ presented a MoS_2_-based phototransistor with asymmetric metal contacts, which not only obtained a fast response rate, but also achieved a high response at the 0 bias. However, there is a question that the dark current of this structure photodetector is quite large, and the light–dark current switching ratio is very small.

On the other hand, many works on the MoS_2_ based self-powered photodetectors have been also reported. A fast and sensitive self-powered photodetector was achieved by using the built-in electric field at the junction of a metal–semiconductor contact^23,24^ or a p–n junction,^25^ which can separate the photogenerated electron–hole pairs under illumination without external bias. Bai *et al.*^26^ fabricated a self-powered photodetector based on MoS_2_/CH_3_NH_3_PbI_3_ heterojunction, which could operate at the 0 bias. However, the photodetector suffered from slow photoresponsivity and lower stability due to the unstable performances of perovskite. Kufer *et al.*^27^ completely passivated MoS_2_ device surfaces with a hafnium dioxide (HfO_2_) layer. Although this method reduced the dark current, the detector wasn't self-powered and can't work without a battery or an external power source. For MS structured photodetector, semiconductor can form the deep depletion region, causing electron–hole pairs were separated by a strong built-in electric-field. In order to improve the performance and reduce the tunneling current from metal to semiconductor, a thin insulator layer can be inserted between semiconductor and metal electrode forming a MIS contact. Thus, not only the optoelectronic performance of photodetector can be improved, but also self-powered device can be obtained by MIS structure.

Herein, a MIS (Pd/Al_2_O_3_/MoS_2_) structured photodetector was fabricated based on MoS_2_. To improve the optoelectronic performance of photodetector, we also fabricated MIS structured Pd/HfO_2_/MoS_2_ photodetector. Significantly, the MIS structured photodetector exhibited obvious photovoltaic effect, enabling their applications as self-powered photodetector worked at zero bias. Due to the ultrathin HfO_2_ insulating layer, this MIS structured photodetector demonstrated as high-efficiency self-powered photodetectors with high responsivity of 538 mA W^−1^. The performance characteristics of the MIS structured photodetectors have largely surpassed those reported for MoS_2_-based photodetectors, exhibiting a self-driven, fast response/recovery time and high responsivity. The results of this paper suggest that the MIS structured photodetectors are promising for constructing high-performance optoelectronic devices.

## Experimental section

### Materials synthesis

The MoS_2_ samples were grown on the Si substrate with a 300 nm SiO_2_ insulation layer by the chemical vapor deposition method, which is similar to previous report.^31,39^ The Si/SiO_2_ substrates were cleaned in acetone, alcohol and deionized water in turn. MoO_3_ (≥99.5% purity) and sulfur (≥99.5% purity) were applied as precursor and reactant materials, respectively. The MoO_3_ powder (10 mg) was placed in quartz boat and located in the center of heating zone of the furnace. A 2 × 2 cm^2^ Si/SiO_2_ substrate used for growing MoS_2_ was face down at top of the MoO_3_ powder. S powder (500 mg) was placed in a separate ceramic boat at the upper stream and heated to 170 °C and carried through Ar flow of 100 sccm. The experiments were implemented at a reaction temperature of 850 °C for 30 min. Finally, the samples were taken out when the furnace naturally cooled down to room temperature.

### Deposition of Al_2_O_3_/HfO_2_ layer by atomic layer deposition (ALD)

An ALD system was applied to coat 0.7 nm Al_2_O_3_/HfO_2_ on MoS_2_ surface. Two solenoid valves controlled the amount of trimethylaluminum (dimethylamino hafnium) and H_2_O vapor. The MoS_2_ substrate was placed in a quartz tube. 50 sccm N_2_ was introduced into the chamber to use as the carrier gas. When deposit the Al_2_O_3_/HfO_2_ film, the system temperature was kept at 120 °C. H_2_O and trimethylaluminum (dimethylamino hafnium) were pulsed into the reaction chamber respectively with pulsing time of 500 ms and followed by 60 s N_2_ purging. After 7 cycles of deposition, the system was cooled down under N_2_ flow. Additionally, AFM imaging on Al_2_O_3_ thin film and HfO_2_ thin film, as shown in Fig. S5,† which illustrates that the two insulating layers are uniformly and continuously covered on the monolayer MoS_2_.

### Device fabrication

Silicon substrate was sequentially cleaned in acetone, alcohol and deionized water for 20 min, and dried by N_2_ flow. Cr electrodes were deposited on top of the substrates by thermal deposition with a shadow mask. As-grown MoS_2_ film was spin-coated with PMMA and dried on a hot-plate. After that, the PMMA-caped MoS_2_ was then submerged in the HF (5%) solution at room temperature for 15 min. The PMMA/MoS_2_ film was lifted from the solution, diluted in DI water, and then transferred onto target substrate with Cr electrodes. The substrate dried on a hot-plate. And the PMMA was removed by acetone vapor. An ALD system was applied to carry out the 0.7 nm insulation layer coating of Al_2_O_3_ (HfO_2_) on MoS_2_ surface. Finally, Pd electrodes were deposited on top of the substrates by thermal deposition with a shadow mask. The schematic diagram of fabricating the MIS structured photodetector is showing in Fig. S2.†

### Device measurements

The surface of the samples was studied by using an optical microscope. Raman and PL spectroscopy measurements were carried with a confocal microscopy (JY-HR800) under 532 nm laser with a power of 20 mW. The spot area of the laser is about 1 mm^2^. The optoelectronic characteristics and the photoresponse properties were measured by a Keithley-4200 SCS. The photocurrent *versus* position curve used 532 nm laser as light source. All electrical and optical properties were measured under the ambient atmosphere.

## Results and discussion

**Fig. 1:**
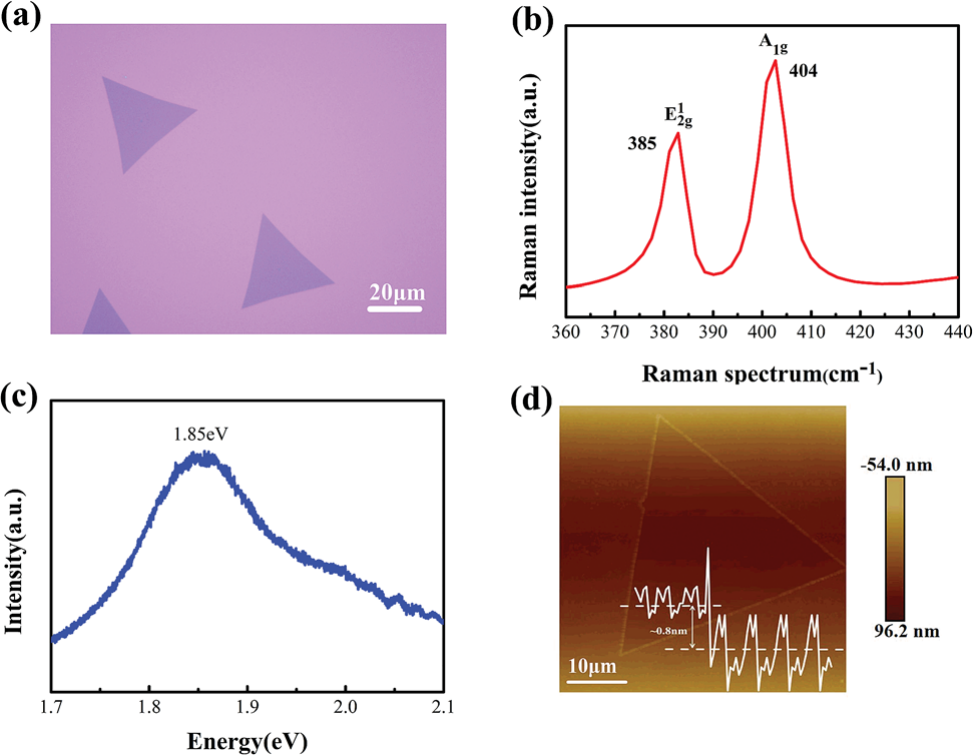
Characterization of the layered MoS_2_ sample. (a) Optical microscope image of CVD-grown typical monolayer MoS_2_ on a 300 nm SiO_2_/Si substrate. (b) Raman spectrum for the monolayer MoS_2_ and (c) PL spectrum for the monolayer MoS_2_. Both Raman and PL experiments measured with a 532 nm excitation laser. (d) Atomic force microscopy image of the as-grown MoS_2_. It shows that the MoS_2_ film is about 0.8 nm thick.

Two-dimensional MoS_2_ was synthesized on the Si substrate with a 300 nm SiO_2_ insulation layer through chemical vapor deposition (CVD) method. The MoO_3_ and sulfur powder were used as the precursor and reactant materials respectively.^29^ It is similar to the method discussed in ref. 30–32 The Optical image of as-grown MoS_2_, as shown in Fig. 1a, displayed a remarkable feature of monocrystalline CVD MoS_2_. Measured with a 532 nm laser, the Raman spectrum of as-grown MoS_2_ presented in Fig. 1b offers a valuable insight into the chemical bonding structures of MoS_2_ and identifies the probable layer number of as-grown MoS_2_.^33^ E_2g_^1^ is an in-plane optical mode, and A_1g_ corresponds to out-plane vibration of the sulfur atom.^34^ A study has been reported that the difference between two peaks relates to the MoS_2_ layers. The frequency difference was 19 cm^−1^ between the two peaks, which illustrated that the as-grown MoS_2_ was monolayer.^35^ The Raman spectrum shown in Fig. S1† a confirmed the phase of the as-grown MoS_2_ without any peaks appearing near the three positions of 152.68 cm^−1^, 226 cm^−1^, 330.29 cm^−1^, which indicated the phase of the as-grown MoS_2_ is 2H phase. The photoluminescence (PL) spectrum of the MoS_2_ sample is shown in Fig. 1c, in which peak was located at about 1.85 eV.^36,37^ Both Roman and PL peaks showed high crystallinity of CVD-grown MoS_2_. Fig. 1d and the inset show a typical AFM topography and the height of the triangular MoS_2_ sample, respectively. The MoS_2_ was measured to be about 0.8 nm thickness in the AFM image, which confirmed its monolayer character.

**Fig. 2:**
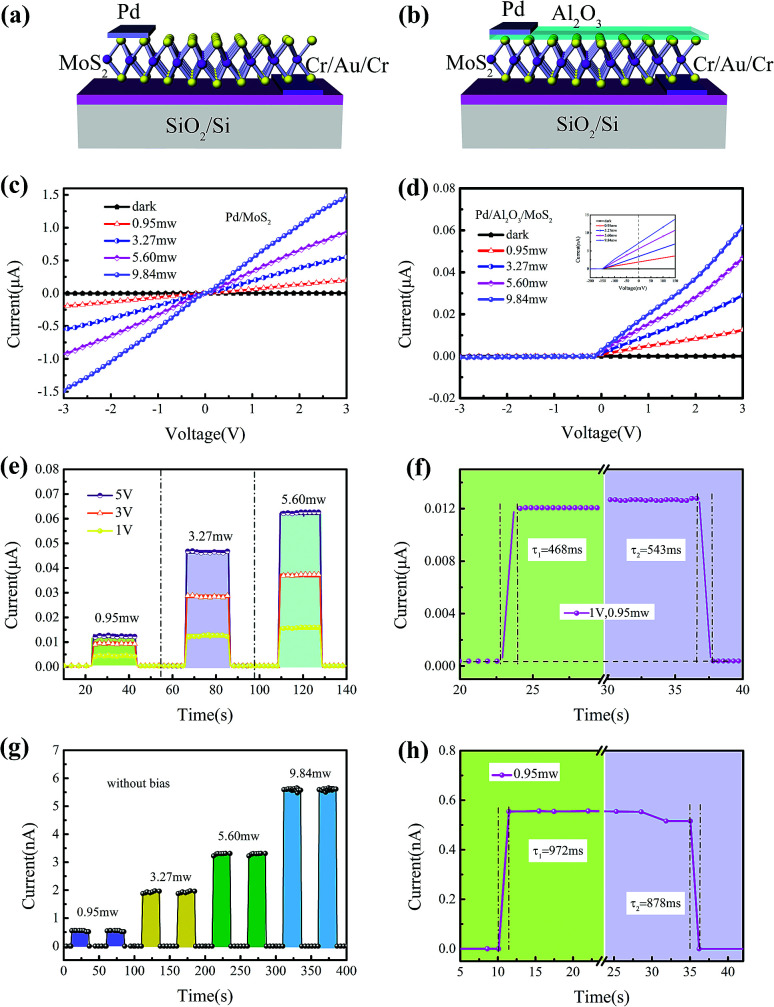
Schematic structure and optoelectronic characteristics of the MS structured Pd/MoS_2_ photodetector and MIS structured Pd/Al_2_O_3_/MoS_2_ photodetector. (a) and (b) Schematic diagrams of MS structure of Pd/MoS_2_ photodetector fabricated on a 300 nm SiO_2_/Si substrate and MIS structure of Pd/Al_2_O_3_/MoS_2_ photodetector, respectively. Current–voltage curves of the device (c) Pd/MoS_2_ MS structure and (d) Pd/Al_2_O_3_/MoS_2_ MIS structure photodetector. The obvious photovoltaic characteristics of MIS structure showed in the inset. (e) and (f) Current–time curves of the MIS structured Pd/Al_2_O_3_/MoS_2_ photodetector under bias. The response/recovery time of 468 ms/543 ms at bias voltage of 1 V. (g) and (h) The self-powered photocurrent responses of the MIS junction with different intensities of illumination. The response/recovery time of 972 ms/878 ms without bias.

Fig. 2a and b show the schematic structure of MS structured Pd/MoS_2_ photodetector and MIS structured Pd/Al_2_O_3_/MoS_2_ photodetector, respectively. To illustrate the effect of the insulator layer at the interface, the performance of MS structured Pd/MoS_2_ photodetector was tested first. The current–voltage characteristics of MS structured photodetector were measured at power intensities of 0, 0.95, 3.27, 5.60 and 9.84 mW, respectively (Fig. 2c). The black line is the dark *I*–*V* curve. The reverse leakage current of the dark curve was measured −2.09 nA at −3 V, which might be a lowered Schottky barrier height (SBH) at the Pd/MoS_2_ interface. Under illumination, the *I*–*V* curves are linear curves, indicating that the Schottky barriers were screened by the charged surface.

Fig. 2d depicts the *I*–*V* curve of MIS contact of Pd/Al_2_O_3_/MoS_2_ under dark condition and light illumination condition with different powers. Under dark, the MIS junction presented an excellent rectifying behavior with a reverse leakage current of −1.39 pA at −3 V. The Al_2_O_3_ insulator layer effectively reduced the leakage current by 3 orders of magnitude compared with the Pd/MoS_2_ contact. When it was exposed to light with different powers of 0.95, 3.27, 5.60 and 9.84 mW, the photo currents were enhanced dramatically. Obvious photovoltaic characteristics of the device can be observed, as shown in the inset. Moreover, the on/off ratio was measured at different power intensities and bias voltages. At the bias voltage of 3 V, the on/off ratio of the MIS device reaches up to 3705. Responsivity can be calculated as following:1
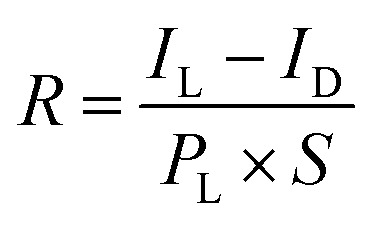
where, *R* is responsivity; *I*_L_ is the current under light illumination, *I*_D_ stands for the current under dark condition, *S* is the active areas, and *P*_L_ represents illumination power. At a light power of 0.95 mW and a bias of 3 V, the Pd/Al_2_O_3_/MoS_2_ MIS structure photodetector exhibited a responsivity of 5.04 A W^−1^.

To analyze the response performance under light illumination, the time-dependent response curves in Fig. 2e are obtained at different bias voltages and different power intensities. According to the previous report,^32^ photocurrent increases continuously with the light power because scattering reduces between photoexcited electrons. The time increasing from 20% to 80% of the equilibrium value from the initial current is the response time of the photodetector, while the time decreasing from 80% to 20% is the recovery time. For this photodetector, Fig. 2f illustrates the response time and recovery time were measured to be 468 ms/543 ms at the bias of 1 V, which shows the faster photoresponse of the photoconductor. Under the light of different powers and a bias of 0 V, the power-dependent photo response curves of the MIS self-powered photodetector are shown in Fig. 2g, which meaned that the MIS structured photodetector can also act as a self-powered photodetector. The photodetector exhibited obvious photoresponse to different light illumination without external bias. Fig. 2h displays that the response/recovery time is 972 ms/878 ms at the bias of 0 V, indicating that the photoexcited carriers can be separated effectively. Its response speed is a little lower than that of under biases. The reason is that the separation of electron–hole pairs and the transfer of photocarrier consumed some time. By comparing, the photocurrent of Pd/MoS_2_/Cr asymmetric electrodes photodetector is higher, indicating that asymmetric metal contacts form a strong built-in electric-field, the separation and transmission of photocarriers can be promoted under light illumination, which is consistent with the lately report.

**Fig. 3:**
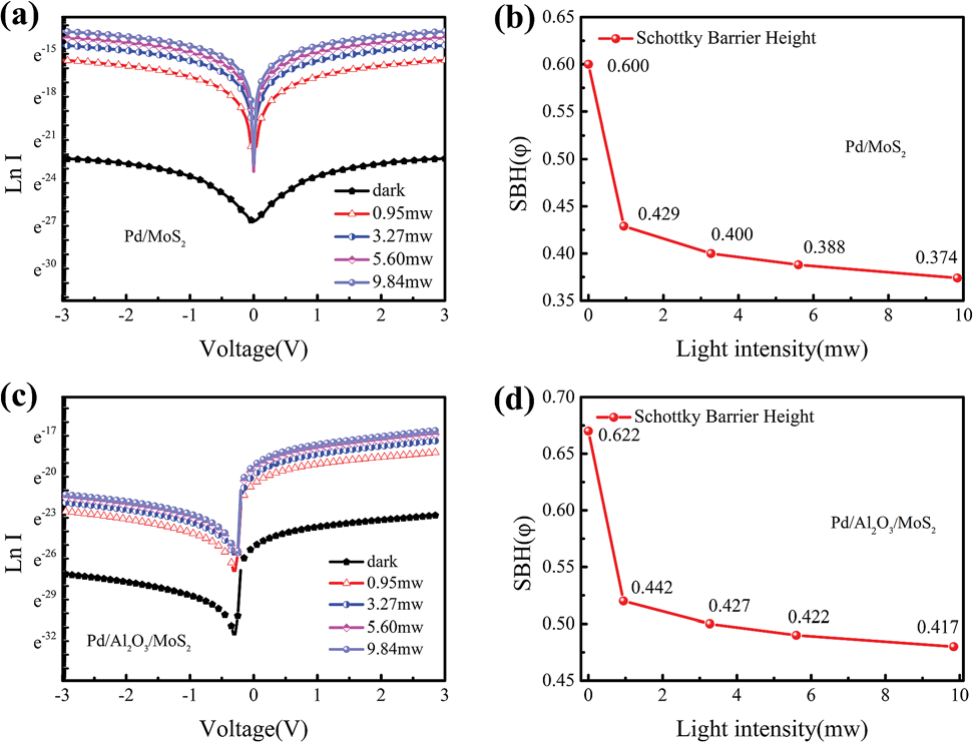
Effect of light intensity on *I–V* curves with the curves in logarithm coordinate of the MS structured Pd/MoS_2_ photodetector and MIS structured Pd/Al_2_O_3_/MoS_2_ photodetector and Schottky barrier height. (a) and (b) Output characteristic on logarithmic scale of the MS structured Pd/MoS_2_ photodetector and calculated Schottky barrier height by using standard thermionic emission theory, respectively. (c) and (d) Output characteristic on logarithmic scale of the MIS structured Pd/Al_2_O_3_/MoS_2_ photodetector and calculated Schottky barrier height by using standard thermionic emission theory, respectively.

Fig. 3a shows the curves of natural logarithm of current *versus* voltage of MS structured photodetector. According to the thermionic-emission theory, Schottky barrier height (SBH) can be calculated, as following:^38^2
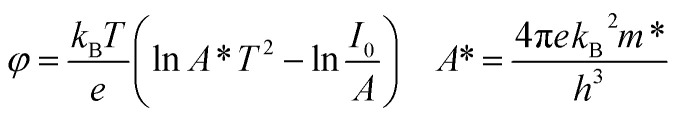
where *φ* is the SBH, *k*_B_ is the Boltzmann's constant, *T* is the temperature, *e* is the electronic charge, A* is the effective Richardson constant, *A* is the contact area, which is the area of the electrode with MoS_2_, m* = 0.45 *m*_0_ is the effective mass of electrons in MoS_2_, and *I*_0_ is extracted from the curves in Fig. 3a. The calculated results of *φ* under different light powers are plotted in Fig. 3b. It presents that the SBH decreases linearly with the increase of light intensity and is independent of external bias.

Fig. 3c shows the curves of natural logarithm of current *versus* voltage of MIS structured Pd/Al_2_O_3_/MoS_2_ photodetector. The calculated results of *φ* under different light powers are plotted in Fig. 3d. As the pictures show, the *φ* of the MIS structured photodetector was much larger than that of the MS structured photodetector. With the increase of light intensity, the *φ* of the MIS structured photodetector were slightly reduced by the photogenerated charges. For a self-powered photodetector, the photocurrent is closely related to the intensity of the built-in electric field and the amount of photogenerated charges. The thin insulator layer can prevent MoS_2_ surface adsorption, hence it can increase the SBH between electrodes and MoS_2_. It is clear that MIS structure photodetector is more suitable to be a self-powered photodetector.

**Fig. 4:**
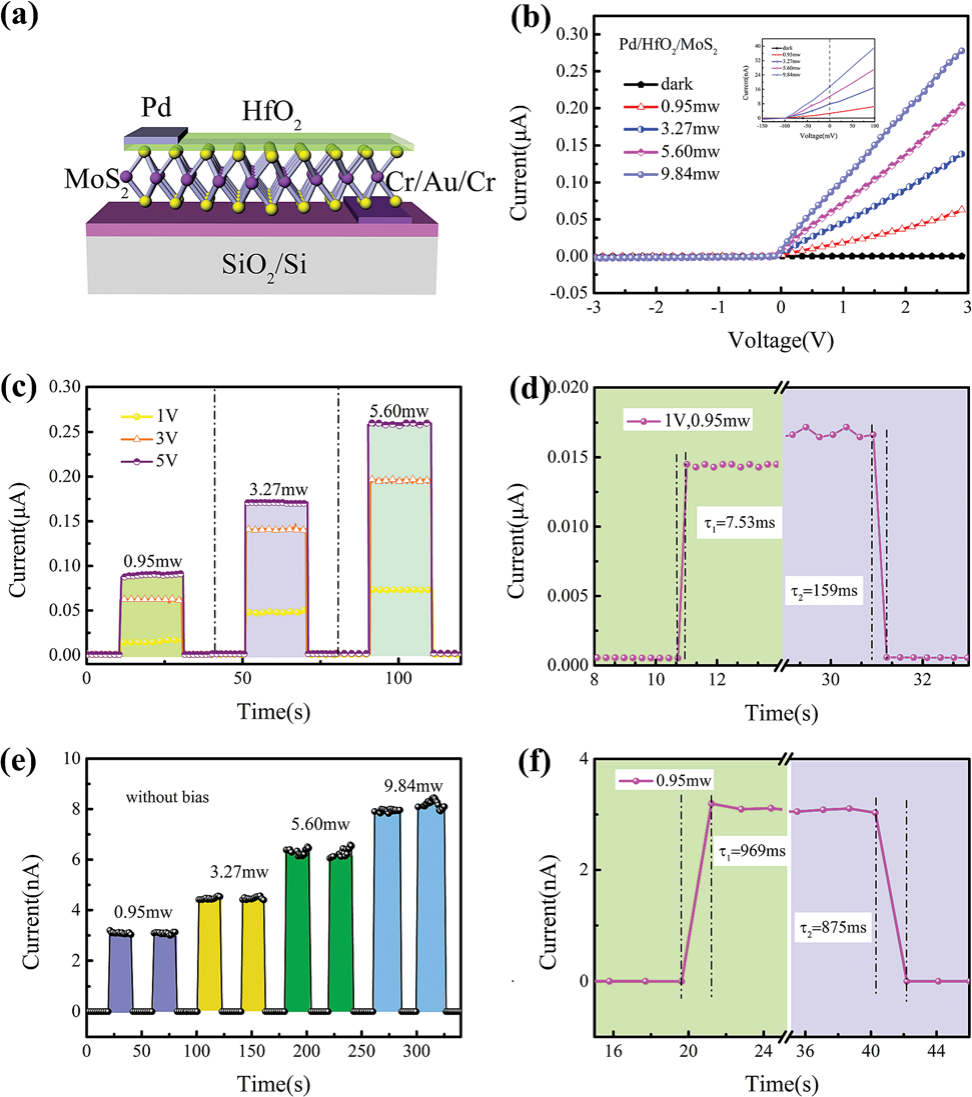
Schematic structure and optoelectronic characteristics of the MIS structured Pd/HfO_2_/MoS_2_ photodetector. (a) Schematic diagram of the MIS structure of Pd/HfO_2_/MoS_2_ photodetector fabricated on a 300 nm SiO_2_/Si substrate. (b) Current–voltage curves of the MIS structured Pd/HfO_2_/MoS_2_ photodetector. The inset shows the photovoltaic effect of the MIS structured Pd/HfO_2_/MoS_2_ photodetector under different 532 nm illumination intensities. (c) and (d) Current–time curves of the MIS structured Pd/HfO_2_/MoS_2_ photodetector under bias. The response/recovery time of 7.53 ms/159 ms at bias of 1 V. (e) and (f) The self-powered photocurrent responses of the MIS junction with different intensities of illumination. The response/recovery time of 969 ms/875 ms without bias.

To optimize the performances of the Pd/Al_2_O_3_/MoS_2_ photodetector, a MIS structured Pd/HfO_2_/MoS_2_ photodetector was further constructed. The schematic structure of the MIS structured Pd/HfO_2_/MoS_2_ photodetector is shown in Fig. 4a. The *I*–*V* curves of the MIS structured Pd/HfO_2_/MoS_2_ photodetector were studied under dark condition and light illumination condition with different powers (Fig. 4b). Under dark, the MIS junction presented an excellent rectifying behavior with a reverse leakage current of −1.01 pA at −3 V bias. The HfO_2_ insulator layer effectively reduced the leakage current by 3 orders of magnitude compared with the Pd/MoS_2_ contact. Under illumination, the photo currents were enhanced dramatically. Obvious photovoltaic characteristics of the device can be observed. At the bias voltage of 3 V, the on/off ratio of the MIS device was measured exceeding 6653. At a light power of 0.95 mW and a bias of 3 V, the MIS structured Pd/HfO_2_/MoS_2_ photodetector exhibits a responsivity of 25.46 A W^−1^. Fig. 4c shows the power-dependent photo response curves of the Pd/HfO_2_/MoS_2_ device with different forward bias of 1 V, 3 V and 5 V under light illumination. The MIS structured photodetector exhibits a clear photo response to the different bias and the photocurrent distinctly increases with the increase of bias. The response/recovery time of this photodetector was 7.53 ms/159 ms at the bias of 1 V (from 20% to 80% of change time), as shown in Fig. 4d, which illustrates that the photoexcited carriers can be separated rapidly.

As presented in Fig. 4e, the photodetector exhibits obvious photoresponse to power light illumination without any external bias. Because the separation and the transfer of electron–hole pairs cost some time, its response speed was a little lower than that of under biases. The response/recovery time of the self-powered photodetector was 969 ms/875 ms at 0 V (Fig. 4f).

**Fig. 5:**
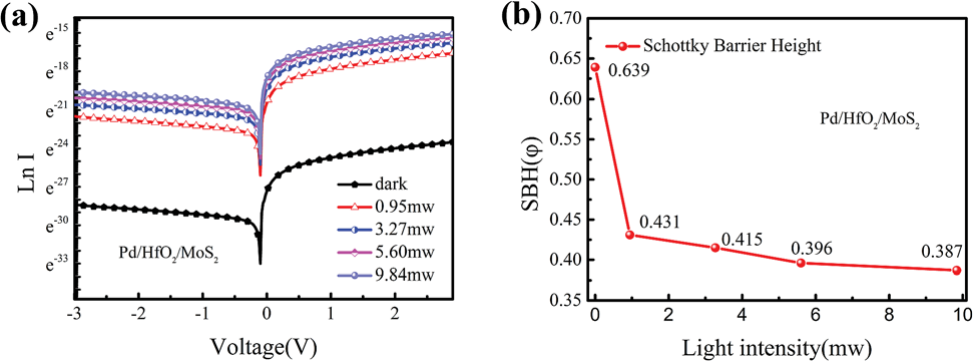
Effect of light intensity on *I–V* curves with the curves in logarithm coordinate of the MIS structured Pd/HfO_2_/MoS_2_ photodetector and Schottky barrier height. (a) Output characteristic on logarithmic scale of the MIS structured Pd/HfO_2_/MoS_2_ photodetector. (b) Calculated Schottky barrier height values of the MIS structured Pd/HfO_2_/MoS_2_ photodetector by using standard thermionic emission theory.

The curves of natural logarithm of current *versus* voltage of MIS structured Pd/HfO_2_/MoS_2_ photodetector are shown in Fig. 5a. Using the thermionic-emission theory, Schottky barrier height (SBH) was calculated. The calculated results of *φ* under different light powers are plotted in Fig. 5b. With the increase of light intensity, the SBH decreases linearly, which is independent of external bias, which also confirmed that the SBH of the MIS structured photodetector is much larger than that of the MS structured photodetector. With the increase of light intensity, the SBH of the MIS structured photodetector was also slightly reduced by the photogenerated charges. This result undoubtedly proves that MIS structured photodetector is indeed more suitable to be a self-powered photodetector.

The above results clearly unveil that the important role of a thin insulation for fabricated the MIS structured MoS_2_ photodetector not only shorten response/recovery time of MoS_2_ based photodetector, but also have a high responsivity and achieve self-driven. The insulator can avoid the contact of MoS_2_ with air, which can reduce the scattering of electrons and increase its electrical transport performance. With the thickness of the insulator increases, the photocurrent of the device also increases, as shown in Fig. S3.† However, when the thickness is increased to 1 nm, the photocurrent is reduced. That's because that if the thickness of the insulator is too thick, it will affect the light absorption of MoS_2_, which can make the photocurrent of the device reduces. And if the thickness of the insulator is too thin, the insulator doesn't function as a tunneling layer, which also can make the photocurrent is relatively small. The photocurrent is maximum when the insulator is 0.7 nm. The performance characteristics of the MIS structured photodetectors in this work have largely surpassed those reported for MoS_2_-based photodetectors, as shown in Table 1, exhibiting a self-driven, fast response/recovery time and high responsivity.

**Table 1:** Performance comparison of the photodetectors with previous works

Ref.	Device	Layer thickness	Optimization	Responsivity	Self-powered mode
**This work**	**Our works: Pd/Al**_**2**_ **O**_**3**_ **/MoS**_**2**_	**Monolayer (CVD)**	**Insert insulator**	**5.04 A W** ^**−1**^	**308 mA W** ^**−1**^
**This work**	**Pd/HfO**_**2**_ **/MoS**_**2**_	**Monolayer (CVD)**	**Insert insulator**	**25.46 A W** ^**−1**^	**538 mA W** ^**−1**^
18	MoS_2_ phototransistor	Monolayer (exfoliation)	—	7.5 mA W^−1^	—
21	Au/MoS_2_/Pd	Multilayer	Asymmetric electrodes	5.07 A W^−1^	100 mA W^−1^
28	Ti/TiO_2_/MoS_2_	5 to 15 nm	Insert TiO_2_	9 A W^−1^	—

**Fig. 6:**
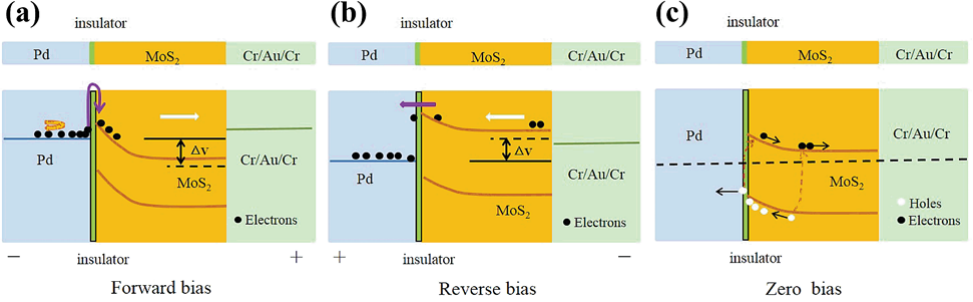
The energy band diagrams of the fabricated MIS structured photodetectors. The energy band diagram of the MIS structured photodetectors (a) with a forward bias (the purple arrow is the tunneling current. The black dash dot line is the Fermi level at thermal equilibrium), (b) with a reverse bias (the purple arrow shows thermionic emission of electrons), and (c) at zero bias, respectively.

For two kinds of the MIS structured devices, the energy band diagrams under illumination are the same. The schematic illustration of the MIS structured photodetectors with sensitivities is shown in Fig. 6. The insulation properties of HfO_2_ better than those of Al_2_O_3_ due to the larger dielectric constant of HfO_2_ than that of Al_2_O_3_. In the dark state, the interface between HfO_2_ will block the transport of carriers. Therefore, the Pd/HfO_2_/MoS_2_ self-powered photodetector has a high Schottky barrier height, causing the dark current of the Pd/HfO_2_/MoS_2_ self-powered photodetector is smaller than that of the Pd/Al_2_O_3_/MoS_2_ self-powered photodetector. When the light irradiates device, more carriers will be blocked at the HfO_2_ interface. Thus, a larger built-in electric field will be formed at the interface, which can facilitate the separation of electron–hole pairs and generate larger photocurrents. Therefore, when the light irradiates the Pd/HfO_2_/MoS_2_ self-powered photodetector, the Schottky barrier height is reduced, the responsivities are improved, and the response time is shortened. The properties of the Pd/HfO_2_/MoS_2_ self-powered photodetector are better than those of the Pd/Al_2_O_3_/MoS_2_ self-powered photodetector.

Here the mechanism for the reduction of the dark current by inserting the insulating layer is explained. Fig. 6a indicates the band alignment schematic diagram of the MIS structured photodetector with a forward bias (a positive bias is applied on the Pd electrode). Under dark, when electrons flow into the Pd electrode through MoS_2_, they can store enough energy under the applied electric field. Therefore, they can very easily tunnel through the insulating layer, which results in a large dark current.^22^ However, the dark current decreased compared with MS structured photodetector due to the blocking of the insulator. When the devices operate under illumination, the photogenerated electrons are generated in MoS_2_ film in large amounts. So, the original existing electrons can be neglected. The photogenerated electrons will be accelerated under the forward bias. Thus, all of the photogenerated electrons will have sufficient energy to flow into the corresponding electrode. The energy band diagram with a reverse bias (a reverse bias is applied on the Pd electrode) shown in Fig. 6b illustrates the photo response process in MIS structured photodetectors. When electrons flow into the Cr electrode from the Pd electrode, they encounter the insulating layer immediately. Most of the electrons haven't enough energy to tunnel through the insulating layer except a small part (the purple arrow) due to without an accelerating process.^23^ The small part of electrons through the interface by thermionic emission, but most of the electrons are blocked on the metal/insulator interface, which obviously reduces the dark current. As a result, the MIS structured photodetectors have a smaller dark current and show a good rectifying behavior.

The photoresponse characteristics of the MIS structured self-powered photodetectors without any external bias can be further understood from the energy-band diagram (Fig. 6c). When the light illuminates at the junction, electron–hole pairs are generated. Then, the photogenerated holes drift toward the Pd electrode tunneling through the insulator layer, and the photogenerated electrons flow to the Cr electrode. In addition, due to the larger dielectric constant of HfO_2_ than that of Al_2_O_3_, the properties of MIS structured Pd/HfO_2_/MoS_2_ photodetector are better than those of the Pd/Al_2_O_3_/MoS_2_ photodetector.

## Conclusions

In conclusion, two kinds of MIS structured self-powered photodetectors of Pd/Al_2_O_3_/MoS_2_ and Pd/HfO_2_/MoS_2_ were fabricated by inserting a thin insulating layer, which demonstrated that inserting the ultrathin insulating layers can optimize the performances of the photodetectors, especially it resulted in fast response/recovery time and self-powered photodetector. Compared with the MS structured photodetector, the MIS photodetectors exhibit good rectifying behaviors and have a smaller dark current due to the selective tunneling current transport. The thin insulating layer not only can increase Schottky barrier height, but also can prevent MoS_2_ surface adsorption. By depositing 0.7 nm insulator layers at the interfaces, the effective SBHs were increased. The properties of the Pd/HfO_2_/MoS_2_ self-powered photodetector are better than those of the Pd/Al_2_O_3_/MoS_2_ self-powered photodetector. The MIS structured Pd/HfO_2_/MoS_2_ photodetector exhibited a high responsivity of 538 mA W^−1^ at the bias of 0 V, and the responsivity can up to 25.46 A W^−1^ under bias. Our findings clarify that the MIS structured MoS_2_ self-powered photodetectors might be a good candidate for potential applications in photodetection, photocatalysis and sensors. Our results lead the way for future application of fast response/recovery time and high responsivity of MoS_2_ detectors and prompt for further investigation in the mechanism of the MIS structured 2D MoS_2_ photodetectors.

## Conflicts of interest

There are no conflicts to declare.

## Supplementary Material

RA-008-C8RA05511D-s001
