# Supplementary material for: Self-powered, high response and fast response speed metal–insulator–semiconductor structured photodetector based on 2D MoS2
Source: RSC Adv. 2018 Aug 6;8(49):28041–7. doi: 10.1039/c8ra05511d (PMC9084249; doi:10.1039/c8ra05511d)
Supplement: RA-008-C8RA05511D-s001 [file RA-008-C8RA05511D-s001.pdf]

Electronic Supplementary Material (ESI) for RSC Advances.
This journal is © The Royal Society of Chemistry 2018

Self-powered, High Response and Fast Response Speed Metal-Insulator-Semiconductor Structured Photodetector based on 2D MoS2

Xinxin Liu[a], Feng Li[a], Minxuan Xu[a], Junjie Qi*[a]

†State Key Laboratory for Advanced Metals and Materials, School of Materials Science and Engineering, University of Science and Technology Beijing, Beijing100083, People's Republic of China.

†These authors made equal contributions to this work.

Corresponding Author

*E-mail: junjieqi@ustb.edu.cn.

Raman measurement for as-grown MoS_2 and atomic force microscopy experiment for the electrodes.

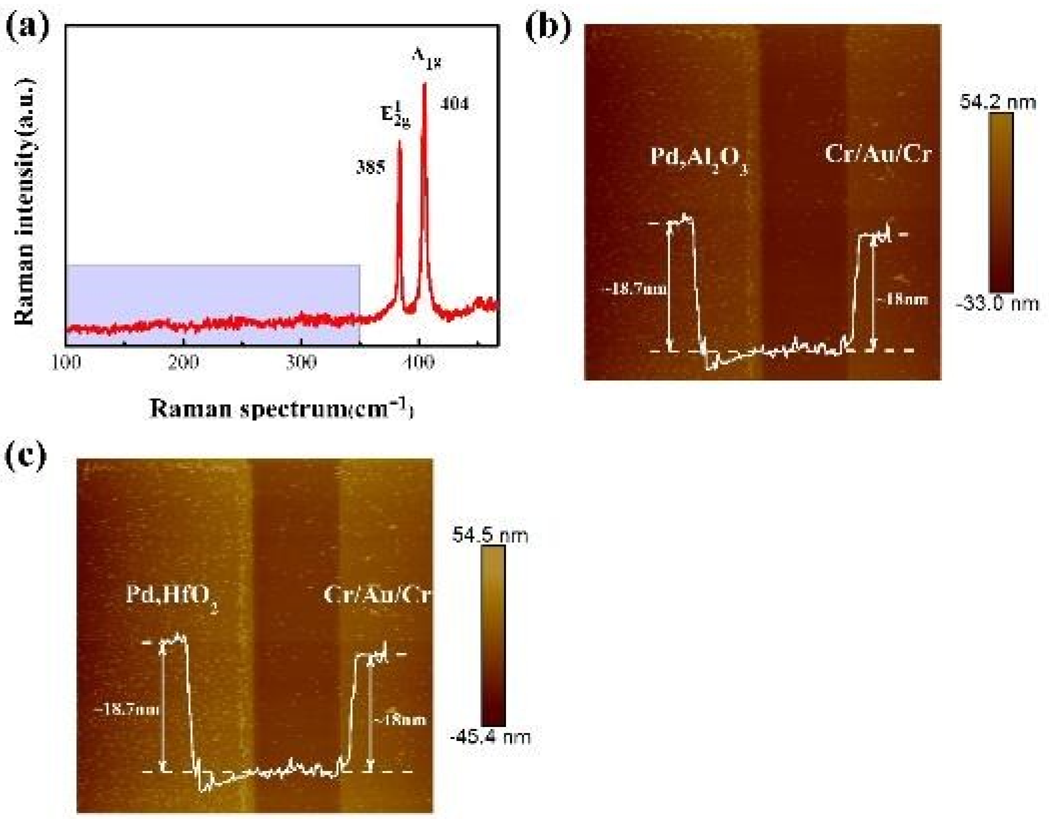

Fig. S1. The Raman spectrum of the MoS_2 and atomic force microscopy image of the electrodes. (a) The Raman spectrum of monolayer 2H phase MoS_2. (b), (c)Atomic force microscopy image of the electrodes of MIS structured $Pd/Al_2O_3/MoS_2$ photodetector and MIS structured $Pd/HfO_2/MoS_2$ photodetector. It shows that the Pd/HfO_2 and Cr/Au/Cr are ~18.7 nm, ~18 nm thick, respectively.

MIS structured photodetectors preparation

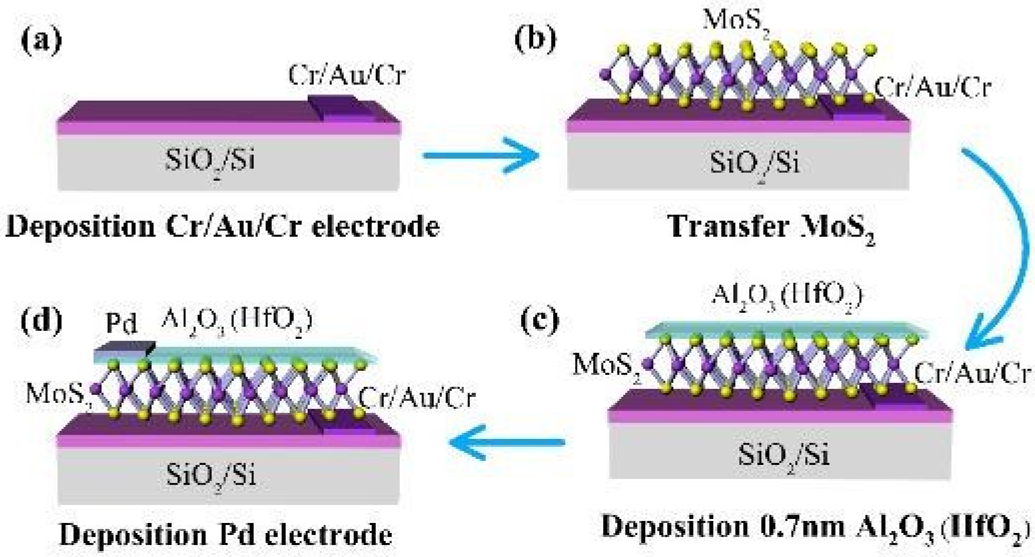

Fig. S2. The schematic diagrams of fabricating progress of the MIS structured photodetectors.

Electrical measurement

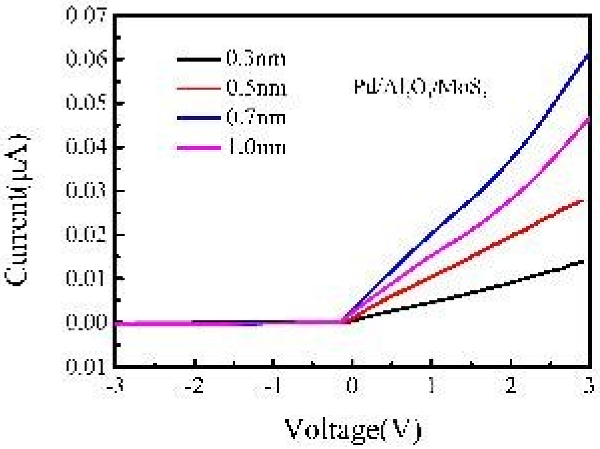

Figure S3. Different thickness of insulutor film on current-voltage curves of the device $Pd/Al_2O_3/MoS_2$ MIS structured photodetector. From picture, when the thickness is 0.7 nm, the photocurrent is the highest. So, we choose 0.7 nm insulator film.

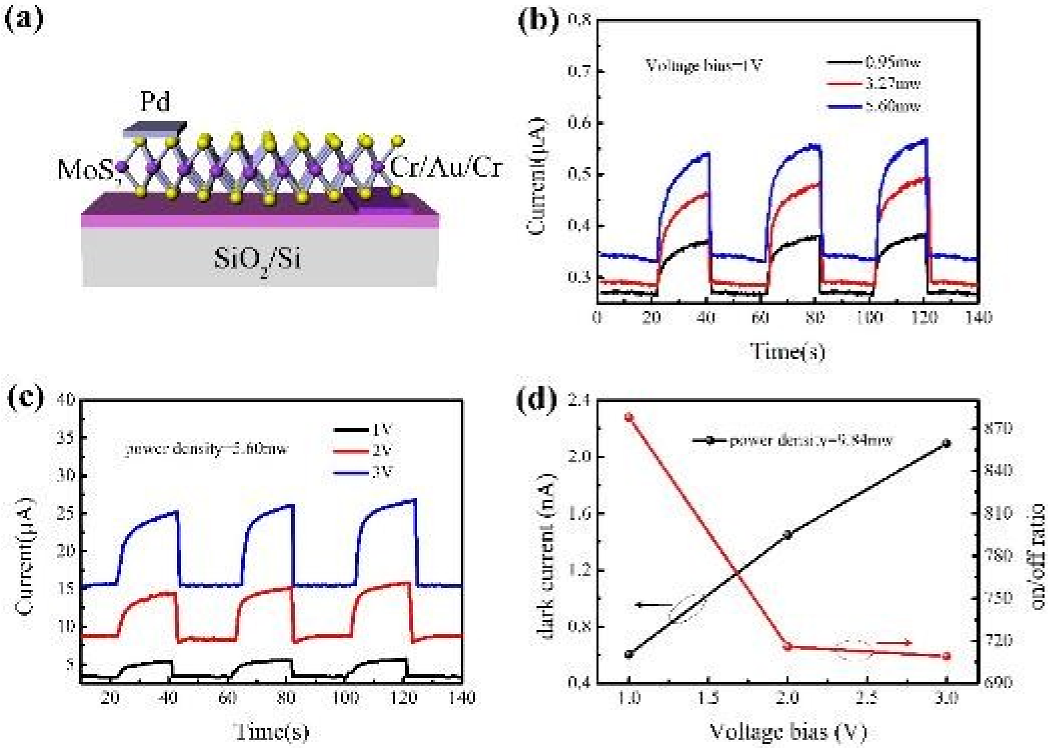

Fig. S4. Schematic structure and optoelectronic characteristic of the MS structured $Pd/MoS_2/Cr$ photodetector. (a) Schematic device structure of the MS structured $Pd/MoS_2/Cr$ photodetector

fabricated on a 300 nm SiO_2/Si substrate. (b) Time-dependent photocurrent response of the MS structured Pd/MoS_2/Cr photodetector under switched-on/off light irradiation with different power at bias voltages of 1V. (c) Time-dependent photocurrent response of the MS structured Pd/MoS_2/Cr photodetector under switched-on/off light irradiation at different bias voltages with 5.60 mw power intensity. (d) Voltage bias-dependent peculiarity of dark current and on/off ratio for this structure with 9.84 mw power intensity, which voltage bias increases, the dark current increase gradually, as well as the responsivity reduce accordingly.

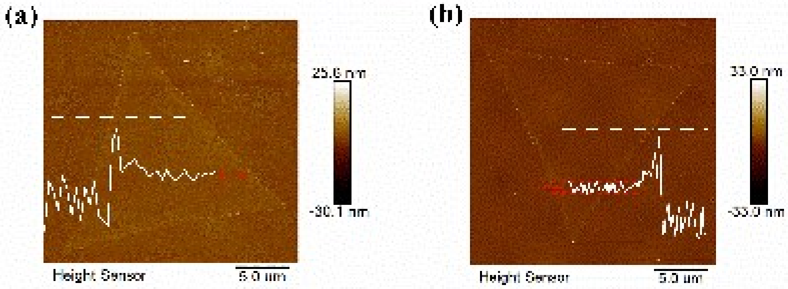

Fig. S5. (a), (b) Atomic force microscopy image of the Al_2O_3 thin film and HfO_2 thin film on the monolayer MoS_2, respectively.
